# Psychological distress and symptom-related burnout in asthma during the COVID-19 pandemic

**DOI:** 10.1007/s10865-023-00412-y

**Published:** 2023-05-25

**Authors:** Margot L. Salsman, Hannah O. Nordberg, Jaxen Howell, Maria Michelle Berthet-Miron, David Rosenfield, Thomas Ritz

**Affiliations:** Department of Psychology, Southern Methodist University, P.O. Box 750442, Dallas, TX 75275-0442 USA

**Keywords:** COVID-19, Pandemic, Asthma, Mental health, Burnout, Symptom burden

## Abstract

People with asthma may be particularly vulnerable to pandemic-related psychological distress, and research is needed to understand the impact of the coronavirus disease 19 (COVID-19) pandemic on their health and well-being. We sought to study the well-being of people with asthma relative to non-asthmatic controls during the COVID-19 pandemic. We also investigated asthma symptoms and COVID-19-related anxiety as potential mediators of distress. Participants completed self-report measures of psychological functioning, including anxiety, depression, stress, and burnout. Controlling for potential confounds, multiple-regression analyses examined differences in psychological health between people with and without asthma. Mediator analyses investigated the role of asthma symptoms and COVID-19-related anxiety in this relationship. 234 adults (111 with asthma, 123 without) participated in an online survey from July to November 2020. During this time, people with asthma reported higher levels of anxiety, perceived stress, and burnout symptoms compared to controls. Elevations in burnout symptoms were found beyond general anxiety and depression (*sr*^2^ = .03, *p* < .001). Reported symptoms typical in both asthma and COVID-19 partially mediated this relationship (P_m_ = .42, *p* < .05). People with asthma reported unique psychological challenges during the COVID-19 pandemic including elevated burnout symptoms. Experience of asthma symptoms played a key role in vulnerability to emotional exhaustion. Clinical implications include increased attention to asthma symptom burden within the context of heightened environmental stress and restricted healthcare access.

**Supplementary Information:**

The online version contains supplementary material available at 10.1007/s10865-023-00412-y.

## Introduction

The health, safety, and economic uncertainties posed by the coronavirus disease 2019 (COVID-19) pandemic have resulted in increased levels of psychological symptoms for populations around the world (Luo et al., 2020). However, the added stress of managing a chronic disease alongside increased vulnerability to severe COVID-19 may enhance this effect for those with underlying conditions (Alonzi et al., 2020; McElroy-Heltzel et al., 2022). For people with asthma, research has indicated that poorer perceived health, increased somatization, and experience of respiratory symptoms could underlie elevated psychological distress, and the overlap of asthma-typical symptoms with those of COVID-19 could result in additional health anxiety (Abrams et al., 2020; Philip et al., 2020). Further, although the full effect of asthma diagnosis on COVID-19 mortality risk remains to be elucidated and could depend on level of asthma control, initial Center for Disease Control (CDC) guidelines advised extra measures for people with asthma to prevent viral infection (Aveyard et al., 2021; Chung, 2021; Mena et al., 2022). While warranted to protect health, strict quarantine measures have led to elevated anxiety and depression in the general population as well as additional barriers to healthcare in those with asthma (Brooks et al., 2020). Indeed, whereas emergency department (ED) admissions for asthma symptoms were lower during the pandemic, a higher rate of transfers from the ED to intensive care for people with asthma during this time could suggest more acute illness upon admission (Nourazari et al., 2021; Sheehan et al., 2021). While the postponement of ED visits during the pandemic may have not been specific to people with asthma, disruptions in routine disease management and immediate intervention for those with asthma can result in life-threatening exacerbations, rendering people with asthma particularly susceptible to long-term consequences from such disruptions. In addition, major trigger factors of asthma exacerbations have become specifically salient during the pandemic. Respiratory infections are among the most common precipitants of a worsening of asthma (Busse et al., 2010, National Heart Lung and Blood Institute/National Asthma Education and Prevention Program, 2020). Further, psychological factors are commonly reported as triggers of asthma symptoms and high levels of psychosocial stress may induce illness exacerbation (e.g., Ritz et al., 2016; Sandberg et al., 2004). Thus, on the most general level, the challenges people with asthma have experienced during the pandemic can be understood under the umbrella of a diathesis-stress model, where the specific vulnerabilities of this chronic respiratory illness interact with the general and illness-specific stressful challenges of this extraordinary life situation to predict adverse mental and physical health outcomes.

Already before the pandemic, people with asthma were more likely to experience anxiety and depression compared to the general population (Opolski & Wilson, 2005; Ritz et al., 2013). Anxiety and depression symptoms are differentially related to physical symptom burden in asthma. Both conditions have been linked to worse asthma control and more healthcare utilization (Schneider et al., 2008), although associations are most consistent for symptoms of depression, including fatigue and low mood (Ahmedani et al., 2013; Kullowatz et al., 2007; Richardson et al., 2008). While the psychological impact of chronic illness is often conceptualized as a “bottom-up” phenomenon (i.e., somatic symptoms cause fatigue and depression), newer models based in computational psychiatry provide a framework to additionally understand psychological symptoms in chronic illness from a “top-down” perspective (Greenhouse-Tucknott et al., 2022). The metacognitive theory of dyshomeostasis (Stephan et al., 2016) posits that detection of “errors” in bodily homeostasis—called “dyshomeostasis”—induces a brain state of fatigue as an initial adaptive response as energy is diverted to regulate bodily processes. However, if dyshomeostasis persists, fatigue gives way to low allostatic self-efficacy (i.e., perceived inability to control one’s symptoms), and then to generalized low self-efficacy, hopelessness, and depression. These psychological symptoms can be expected to exacerbate the patient’s asthma and/or result in decreased motivation to attempt to control symptoms, which, if not addressed, may underlie the link between depression and reduced asthma control.

Interestingly, some studies have found increased prescriptions for inhaled and oral corticosteroids and may suggest that adherence to asthma control measures improved at the start of the COVID-19 pandemic (for a review, see Skene & Pfeffer, 2021). However, recent studies investigating self-reported asthma symptoms have shown that people with asthma more often rated their symptoms as worse since the start of the pandemic—particularly if they had contracted the virus themselves—and that this impacted their psychological well-being (Gomes et al., 2023; Muntean et al., 2023). Unfortunately, the spike in psychopathology during the COVID-19 pandemic has persisted despite the availability of vaccines and rollback of COVID-19 restrictions—an effect particularly strong in those whose physical health was impacted by COVID-19 (Daly and Robinson, 2023). Further, the high likelihood of future pandemics and the strong link between psychological symptoms and asthma control lends a particular urgency to research dedicated to understanding differences in psychological distress in those with asthma during this time (Dodds, 2019).

The current study investigated between-group differences in people with asthma and a non-asthmatic control group to improve understanding of how asthma specifically impacted various domains of psychological well-being during the pandemic. Given that the COVID-19 pandemic can be expected to pose a particular physical and mental burden to people with asthma, we proceeded with the following aims: (1) Examine various domains of psychological distress in people with asthma while controlling for the experience of COVID-related hardships and years of education, (2) determine whether such effects exist beyond general anxiety and depression, and (3) investigate whether scores of perceived COVID-19 vulnerability, experience of COVID-19 symptoms, or symptom-related worry mediate differences in different domains of distress between asthma and controls. Additionally, we sought to determine whether asthma severity and/or control would affect the levels of COVID-19-related psychological distress in asthma.

## Study design and methods

### Study population

Participants completed an online survey administered from July to November 2020 (3–7 months after implementation of regional COVID-19-restrictions). Participants were recruited from a Southern Methodist University research database that oversampled for asthma as well as social media advertisements. All were located within the Southern U.S. Recruited participants were invited by email to complete a RedCap survey exploring lifestyle changes experienced by adults during the COVID-19 pandemic. They were also required to e-sign an informed consent before survey initiation. Invitations were addressed to persons both with and without asthma to avoid motivational biases for reporting an asthma diagnosis. As incentive for participation, participants were able to enter their email address into a raffle for ten $50 gift cards. Of 261 total surveys, data from 234 participants were eligible for analysis at the end of data collection (see the data analysis section for exclusions). 111 of these endorsed a current physician’s diagnosis of asthma. Asthma was categorized as “intermittent” or “persistent” severity based on symptom and medication self-report (National Heart Lung and Blood Institute/National Asthma Education and Prevention Program, 2020). Asthma history, including date of initial physician’s diagnosis and current asthma medications, was collected. However, persistent asthma was not subcategorized into mild, moderate, or severe, because asthma management had been initiated, and/or we were unable to objectively confirm reports of medications required to achieve asthma control. The study was preregistered on the Open Science Framework (osf.io/zt5kg/) and approved by the Southern Methodist University Institutional Review Board (Protocol #H20-103-RITT).

### Measures

#### Domains of psychological distress

Participants completed the following measures to assess various domains of psychological distress: Fear of COVID-19 scale (FCV-19S) (Winter et al., 2020), Patient Health Questionnaire 4 (PHQ-4) capturing general anxious/depressive symptoms (Kroenke et al., 2009), Perceived Stress Scale 4 (PSS-4) measuring of acute stress (Cohen et al., 1983), Impact of Event Scale-Revised (IES-R) assessing pandemic-related stress (Christianson & Marren, 2012), and the Brief Trier Inventory of Chronic Stress (brief TICS) to assess chronic stress (Petrowski et al., 2020). The Maslach Burnout Inventory-General Survey (MBI-GS), a validated measure of burnout, was also administered with instructions modified to expand the scope to feelings towards “jobs, volunteer work, studies, and/or daily routine” in the last three months (Maslach et al., 1986). The MBI-GS consists of three subscales—Emotional Exhaustion (feeling overextended and drained of one’s emotional resources); Cynicism (feeling indifferent); and reverse-scored Efficacy (satisfaction with accomplishments) (Aronsson et al., 2017), each of which was analyzed separately. The retrospective timeframe of each measure fell within the period of government-ordered COVID-19 restrictions.

#### COVID-19 symptom experience

Surveys queried participants’ experience with COVID-19 and asthma symptoms since the beginning of the pandemic. Eleven items reflected the CDC’s list of primary COVID-19 symptoms and partly overlapped with typical asthma symptoms (Abrams et al., 2020; Center for Disease Control, 2021). For thoroughness, “wheezing” and “chest tightness” were added to reflect symptoms typical of asthma (see Table S1 for full symptom list). This ad-hoc 13-item measure showed excellent internal consistency (Cronbach’s α = 0.92, mean interitem correlation *r* = 0.47). Upon endorsement of a symptom, participants were asked for symptom severity (1 = “Mild” to 5 = “Severe”). They then rated their level of worry that this symptom could indicate COVID-19 (0 = “Not at all worried” to 4 = “Extremely worried”). Exploratory factor analysis (maximum likelihood estimation followed by oblique Promax rotation) showed that items fell into two correlated factors (*r*(232) = 0.63, *p* < 0.001): one reflecting symptoms typical in both asthma and COVID-19 (“asthma-typical”; shortness of breath, chest tightness, cough, wheezing, fatigue, muscle/body aches; α = 0.90) and the other consisting of symptoms typical in viral illness but not asthma (“illness-typical”; fever or chills, headache, loss of taste/smell, sore throat, congestion/runny nose, nausea/vomiting, diarrhea; α = 0.84) (Table S1).

To investigate whether the effect of symptom severity on psychological distress would be magnified by symptom worry, the interaction of COVID-19 symptom experience and symptom-related worry was calculated as the product of Symptom Experience by Symptom Worry scores with each variable centered at their respective means.

#### Perceived COVID-19 vulnerability

Participants were asked if they considered themselves more at risk of severe COVID-19 (i.e., resulting in complications or death) than the general population (1 = “No more at risk” to 3 = “Yes, much more at risk”). Participants were also asked about their perceived likelihood of personally contracting COVID-19 in the next two months (0 = “Not at all likely” to 10 = “Very likely”).

To investigate whether the effect of perceived vulnerability on psychological distress would be magnified by the likelihood of contracting COVID-19, an interaction variable representing overall “Perceived COVID-19 Vulnerability” was calculated as the product of Perceived COVID-19 Severity and Perceived Likelihood of COVID-19 with each variable centered at their respective means.

#### Asthma control

Asthma control was measured by the 5-item Asthma Control Test (ACT) (Schatz et al., 2006). Items queried functional impairment, perceived asthma symptoms, and medication use. Scores range from 0 to 25 with higher scores indicating higher levels of control over symptoms.

#### Pandemic-specific adverse events

Participants were asked to indicate whether they had experienced a variety of pandemic-specific adverse events indicated by literature to be particularly stressful (Mousavi et al., 2020). This ad-hoc item list (see Supplementary Materials) contained 30 items querying experience of events such as loss of employment, loss of home, and loss of a close friend or family member to COVID-19. Because some events were rarely if ever endorsed by our sample (e.g., “became homeless”), items were included as control variables if they were endorsed by > 10% of the sample. This left 8 events suitable for inclusion in the model (see Table 3).

#### Exacerbation frequency

To query worsening of asthma symptoms during the pandemic, participants were asked if they experienced an exacerbation since being affected by COVID-19 restrictions on a 5-point scale (“no” = 0 to “yes, 4 + times” = 5). If they endorsed at least one exacerbation, participants were asked how this compared to exacerbation frequency before COVID-19 restrictions (“Much less than normal” to “Much more than normal”).

### Procedure

Participants completed an online battery that surveyed demographics, pandemic-specific adverse events, and recent life changes as well as measured physical health, psychological health, and health behaviors during the COVID-19 pandemic. Participants with asthma were provided an additional battery of asthma-specific surveys. To reduce missing data, a maximum of three follow-up reminders containing a unique link to their partially completed survey were sent by e-mail to partial survey completers at two-week intervals during data collection.

### Data analysis

Statistical analyses were performed using SPSS-25 (IBM Corp, 2017). Participants missing > 50% of all data were excluded (10.3% of total sample; 3.5% of asthma, 15.8% of control). This left 234 out of 261 total surveys eligible for analysis. For eligible participants, individual items on scales with < 50% missing data were imputed using thirty rounds of multiple imputation. Imputation was performed separately for those with and without asthma since participants with asthma had additional measures that were related to their asthma (10.8% and 11.4% of surveys in asthma and control groups had missing data and hence required imputation, respectively). Means, standard deviations (SD), and frequencies were calculated for questionnaire scores. Internal consistency of the scales was compared to prior literature by calculating Cronbach’s α and mean inter-item correlations. No outliers (defined as > 3 SD from the mean) were identified. All variables were tested for skewness and kurtosis, and continuous variables used as predictors or mediators were centered at their respective mean. COVID-19 symptom experience, asthma-typical subscale, and infection-typical subscale scores were skewed and were natural-log transformed. This resulted in skewness < 1 for all variables.

Differences between asthma and control groups on demographic variables, work status, and rates of pandemic-specific adverse events were investigated using original unimputed data. Multiple regression (MR) analyses (one model for each measure of psychological distress) assessed the effect of asthma group on psychological distress during the COVID-19 pandemic (Aim 1). PHQ-4 scores were added to significant models in follow-up analyses to assess for significant group differences over and above general anxiety and depression (Aim 2).

In each analysis, we controlled for demographic variables that have been linked to asthma outcomes (age, gender, White versus Non-White race, and years of education (Pate et al., 2021), as well as endorsement of pandemic-specific adverse events, to account for the impact of particularly stressful life events that could have contributed to health status during the pandemic.

**Fig. 1:**
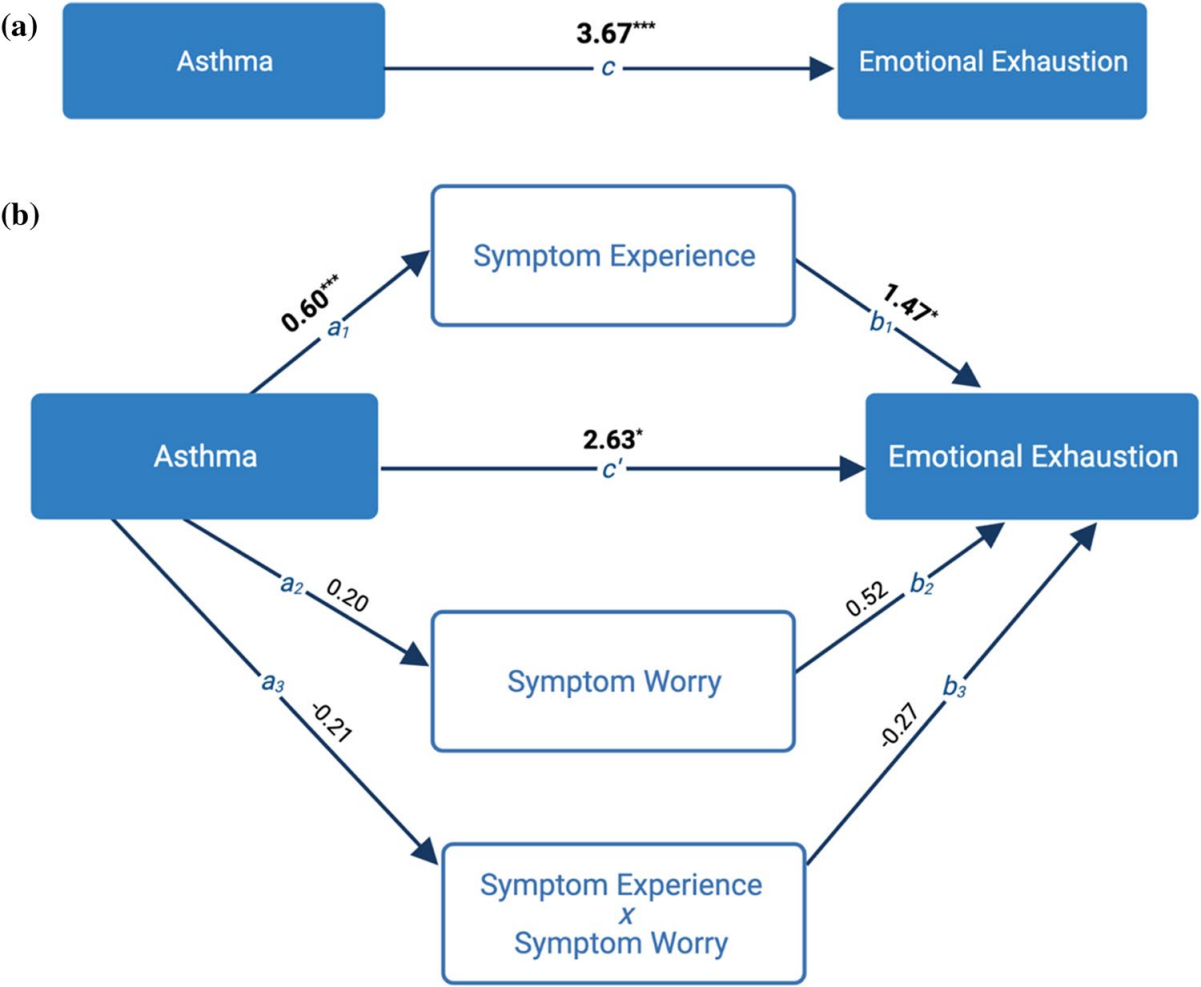
Asthma and COVID symptom report as a mediator of the relationship between asthma and increased pandemic-related emotional exhaustion. **A** The main effect of asthma on pandemic-related emotional exhaustion, controlling for demographics and pandemic-specific adverse experiences. On average, people with asthma scored 3.67 points higher on the MBI-emotional exhaustion scale than individuals without asthma. ***p < .001. **B** Mediation effect of symptom experience, symptom worry, and the interaction of symptom experience by symptom worry on the relationship between asthma and emotional exhaustion. The main effect of average levels of symptom experience significantly mediated the effect of asthma on emotional exhaustion (P_m_ = .24), but average levels of symptom worry and the interaction of symptom experience by symptom worry did not. *p < .05, ***p < .001

Mediator analyses, controlling for demographics, pandemic-specific adverse events, and PHQ-4 scores, investigated two mediators of the relationship between asthma group and psychological distress, each in a separate analysis: (1) Perceived COVID-19 Vulnerability (modeled by Perceived COVID-19 Severity, Perceived Likelihood of COVID-19, and their interaction), and (2) anticipated COVID-19 symptom experience (modeled by anticipated COVID-19 Symptom Severity, Symptom Worry, and their interaction) (Aim 3; Fig. 1B). Significance of mediated pathways was tested with R*Mediation open-source software (Tofighi & MacKinnon, 2011).

Regression coefficients and significance testing for all analyses were derived from pooled MI analyses. In sensitivity analyses, we repeated the analyses outlined above using the non-imputed data set. For *p*-values that meet conventional levels of significance (i.e., *p* < 0.05), we report both the raw *p*-value and whether that test survived correction for the False Discovery Rate (FDR). The FDR critical value was calculated for the fifteen MR analyses investigating our primary aims (Benjamini & Hochberg, 1995; van Ginkel & Kroonenberg, 2014).

With our sample of 234 participants, a post-hoc G*power analysis revealed that our final multiple regression would have 0.94 power to detect a small-to-medium effect size assuming 15 predictors (*f*^2^ = 0.05, α = 0.05) (Faul et al., 2007; Goodwin et al., 2003; Ye et al., 2021). This effect size is consistent with effect sizes obtained in the prior literature on psychological symptoms in asthma.

## Results

### Sample description

The final sample of 234 participants (111 with asthma, 123 without asthma) was predominately white (76.1%), middle class (*M* = 6.12 out of 10, MacArthur Scale of Perceived Social Status, see Adler et al., 2000), female (82.5%), and college-educated (16.1 ± 2.4 years of school) with a mean age of 55.7 ± 9.5 years. One participant (0.4%) reported a current diagnosis of COVID-19 and three (1.3%) reported a past diagnosis with full recovery. Participants with asthma represented all levels of asthma control, with 61.3% of them scoring as “well controlled” asthma (ACT > = 20) and 74.8% meeting criteria for persistent asthma according to symptoms and medications (Table 1) (National Heart Lung and Blood Institute/National Asthma Education and Prevention Program, 2020). Tests of group differences revealed that the asthma group had a higher proportion of females (89.2%) than the non-asthma group (76.4%; χ^2^(1) = 6.58, *p* = 0.010). Also, the event of “death of friend or family member due to COVID-19” was higher in the asthma group (16.2%) than in the control group (5.7%); χ^2^(1) = 6.77, *p* = 0.009). No significant association was found between asthma group and any other demographic variable nor pandemic-related adverse event (Table 1).

**Table 1:** Demographic and health-related characteristics of survey respondents along with reported experience of pandemic-specific adverse events

	All (*N* = 234)	Asthma group (*n* = 111)	Control group (*n* = 123)
Age (years)			
*M* ± *SD*	55.7 ± 9.5	54.4 ± 9.7	56.8 ± 9.1
Range	31–79	31–77	31–79
Gender* (N, %)			
*Female*	193, 82.5	99, 89.2	94, 76.4
Male	41, 17.5	12, 10.8	29, 23.6
Race (*N,* %)			
*White*	178, 76.1	81, 73.0	97, 78.9
Hispanic	20, 8.5	10, 9.0	10, 8.1
Black/African-American	17, 7.3	10, 9.0	7, 5.7
Asian	5, 2.8	2, 1.8	3, 2.4
Native American	7, 3.0	6, 5.4	1, 0.8
Years of education (*M* ± *SD*)	16.1 ± 2.4	16.3 ± 2.7	16.0 ± 2.2
Perceived SES (1–10) (*M* ± *SD*)	6.12 ± 1.9	6.0 ± 1.9	6.2 ± 1.9
Employed full-time (*N,* %)	154, 65.8	74, 66.7	80, 65.0
Asthma severity (*N,* %)			
Persistent	–	83, 74.8	–
*Intermittent*	–	28, 25.2	–
Asthma medications (*N*, %)			
SABA bronchodilators	–	77, 32.9	–
LABA bronchodilators	–	40, 17.1	–
Anticholinergic bronchodilators	–	73.0	–
Inhaled corticosteroids	–	53, 22.6	–
Systemic corticosteroids	–	00.0	–
Leukotriene modifiers	–	23, 9.8	–
IgE inhibitors	–	41.7	–
Antihistamines	–	93.8	–
Unmedicated	–	87.2	–
Pandemic-specific adverse events (*N,* %)			
Loss of employment	54, 23.1	28, 25.2	26, 21.1
Inability to pay bills	48, 20.5	28, 25.2	20, 16.3
Child in home needing care	37, 15.8	21, 18.9	16, 13.0
Increase in home conflict	51, 21.8	27, 24.3	24, 19.5
Relocated home	15, 6.4	8, 7.2	7, 5.7
Improvise living conditions	38. 16.2	22, 19.8	16, 13.0
Unable to get food	24, 10.3	13, 11.7	11, 8.9
Death of close friend or family member**	25, 10.7	18, 16.2	7, 5.7
Asthma control test (*N*, %)			
*Well controlled* (25–20)	–	68, 61.3	–
Not well controlled (19–16)	–	28, 25.2	–
Poorly controlled (< 16)	–	15, 13.5	–

*M* Mean, *SD* Standard deviation, *SABA* Short-acting b-adrenergic, *LABA* Long-acting b-adrenergic

Reference group in italics

Bolded represents a significant difference in values between asthma and control groups, *p < .05, **p < .01

### Aims 1, 2. Differential psychological distress

#### Asthma as a predictor of distress

After controlling for demographics and experience of pandemic-specific adverse events, asthma group (asthma vs. control) explained significant variance in three domains of psychological distress. People with asthma reported elevated anxiety symptoms (PHQ-4 Anxiety; *b* = 0.53, *t*(220) = 2.13, *p* = 0.034, *sr*^2^ = 0.02), elevated perceived stress (PSS-4; b = 0.84, *t*(220) = 2.04, *p* = 0.041 *sr*^2^ = 0.01), and elevated emotional exhaustion (a subscale of the MBI-GS) (b = 4.89, *t*(220) = 3.95, *p* < 0.001, *sr*^2^ = 0.06) during the pandemic (Table 2). Of these domains of psychological distress, only the *p-*value for the association of asthma group with emotional exhaustion survived correction for the FDR.

**Table 2:** Descriptive statistics and multiple linear regression (MR) models for the association of asthma group (asthma vs. non-asthmatic control group) with each measure of psychological distress

	Asthma group (*n* = 111)		Control group (*n* = 123)		MR models			
	*M*	*SD*	*M*	*SD*	B [95% CI]	*SE*	*t*	*p-*value
**PHQ-4 anxiety**	2.91	2.00	2.09	1.85	**0.53 [0.04, 1.01]**	0.25	–	2.13^*^
PHQ-4 depression	2.21	1.73	1.88	1.71	0.19 [− 0.26, 0.63]	0.23	0.83	–
**PSS-4**	7.34	3.14	6.07	3.36	**0.84 [0.33, 1.64]**	0.41	–	2.04^*^
IES-R	11.01	5.52	10.05	5.96	0.39 [− 1.13, 1.91]	0.78	0.50	–
Brief TICS	23.08	6.86	20.94	7.41	1.26 [− 0.53, 3.06]	0.92	1.38	–
FCV-19S	17.94	6.37	16.62	6.06	0.75 [− 0.97, 2.46]	0.87	0.85	–
**MBI-GS Emotional Exhaustion**	23.43	9.66	17.76	8.27	**4.89 [2.47, 7.32]**	1.24	–	3.95***
MBI-GS Cynicism	20.27	8.18	18.33	8.57	1.16 [− 1.12, 3.45]	1.16	1.00	–
MBI-GS Efficacy	30.91	7.40	31.82	7.72	− 0.62 [− 2.61, 1.37]	1.02	− 0.61	–

*MR* multiple linear regression, *PHQ-4* 4-item Patient Health Questionnaire-4, *PSS-4* Perceived Stress Scale, *IES-R* Impact of Event Scale-Revised, *TICS* Trier Inventory of Chronic Stress, *FCV-19S* Fear of COVID-19 scale, *MBI-GS* Maslach Burnout Inventory-General Survey, *SE* standard error

Bolded values are significant at *p < .05, ***p < .001

Only the *p-*value for the association of asthma group with MBI-GS emotional exhaustion scores remained significant after correction for multiple tests

All analyses controlled for demographics and pandemic-related events (see Table 3 for the list of control variables)

**Table 3:** MR analyses of the association of asthma group (asthma vs. non-asthmatic control) with emotional exhaustion symptoms as measured by the Maslach Burnout Inventory-General Survey. All analyses controlled for demographic variables and endorsement of pandemic-related adverse events

Effect	B [95% CI]	*SE*	*t*	*p-*value^d^	% Variance^e^
*Main effects*					
**Intercept**	**10.96 [1.84, 20.07]**	4.65		2.36*	1.15
**Asthma group**^a^	**3.67 [1.75, 5.58]**	0.98		3.75***	3.35
*Control variables*					
**PHQ-4—anxiety**	**2.02 [0.92, 2.34]**	0.36		5.08***	6.40
**PHQ-4—depression**	**1.63 [1.24, 2.79]**	0.40		4.51***	5.06
Age (years)	− 0.08 [− 0.18, 0.30]	0.05	− 1.41	–	0.48
Gender^b^	− 0.92 [− 3.40, 1.57]	1.27	− 0.72	–	0.13
Race^c^	0.81 [− 1.52, 3.15]	1.19	0.68	–	0.12
Years of education	0.28 [− 0.10, 0.66]	0.19	1.46	–	0.50
*Pandemic-specific adverse events*					
Loss of employment	− 0.30 [− 2.53, 1.93]	1.13	− 0.26	–	0.02
Inability to pay bills	− 1.57 [− 4.25, 1.11]	1.37	− 1.15	–	0.30
Child in home needing care	− 0.01 [− 2.75, 2.73]	1.40	− 0.01	–	0.00
Increase in home conflict	− 0.21 [− 2.69, 2.28]	1.27	− 0.16	–	0.01
Relocated home	2.17 [− 1.71, 6.04]	1.98	1.10	–	0.27
Improvise living conditions	0.53 [− 2.18, 3.24]	1.38	0.38	–	0.03
Unable to get food	− 3.07 [− 6.47, 0.32]	1.73	− 1.77	–	0.72
Death of close friend or family member	− 2.17 [− 5.31, 0.96]	1.60	− 1.36	–	0.42

*MR* multiple linear regression, *PHQ-4* 4-item Patient Health Questionnaire-4, *PSS-4* Perceived Stress Scale, *IES-R* Impact of Event Scale-Revised, *TICS* Trier Inventory of Chronic Stress, *FCV-19S* Fear of COVID-19 scale, *MBI-GS* Maslach Burnout Inventory-General Survey, *SE* standard error

^a^0 = non-asthmatic control, 1 = asthma.

^b^0 = female, 1 = male.

^c^0 = White, 1 = Not white

^d^Bolded values are significant at *p < .05, **p < .01, ***p < .001.

^e^% Variance = percent variance over and above other variables

Aim 2 examined whether asthma group remained a significant predictor of distress after controlling for the depression and anxiety subscales of the PHQ-4. Since emotional exhaustion was the only distress domain that was significantly related to asthma group after correction for FDR in Aim 1, it was the only distress domain that was tested in Aim 2. Analysis of emotional exhaustion showed that it remained significantly elevated in asthma even after controlling for general depression and anxiety symptoms (b = 3.67, *t*(218) = 3.95, *p* < 0.001, *sr*^2^ = 0.03) (Table 3).

### Aim 3. Mediator analysis

Since Emotional Exhaustion was the only domain of distress that was significantly elevated among individuals with asthma, the analyses examining the mediators of elevated distress in asthma were only performed on this outcome.

#### Perceived COVID-19 vulnerability as mediator of asthma group differences in emotional exhaustion

COVID-19 vulnerability did not significantly mediate differences in emotional exhaustion scores between those with asthma and those without asthma (a*b = 0.03, 95%CI = [− 0.14, 0.25]) (Table S2).

#### COVID-19 symptom experience as mediator

The main effect of experiencing symptoms was a significant partial mediator of the relationship between asthma group and elevated levels of emotional exhaustion during the pandemic, with the proportion mediated (P_m_) indicating that 24% of the effect was mediated by experiencing symptoms (a*b = 0.88, 95%CI = [0.004, 1.95], *p* < 0.05, P_m_ = 0.24) (Fig. 1B, Table S3). However, the interaction of Symptom Experience by Symptom Worry did not significantly mediate differences in emotional exhaustion scores between those with asthma and those without asthma, nor did symptom worry alone.

#### Associations with asthma severity and control

In analyses including only participants with asthma, and controlling for anxiety and depression symptoms, asthma severity (categorized as persistent or intermittent) did not significantly predict differences in emotional exhaustion (b = 0.17, *t*(220) = 0.10, *p* = 0.92), nor did the continuous ACT score, though the negative relation between asthma control and emotional exhaustion (i.e., more asthma control being related to less emotional exhaustion) did show a trend towards significance (b =− 0.34, *t*(220) =− 1.94, *p* = 0.053) (Table S4).

### Exploratory analyses

In addition to our main analyses, we were interested in determining whether symptoms worsened in this sample during the pandemic. Of the asthma respondents who experienced ≥ 1 exacerbation (29.2%) during this time, 60.6% reported an increase in exacerbation frequency versus before COVID-19 restrictions, 27.3% reported no change, and 12.2% reported decreased frequency. To investigate the validity of the “asthma-typical” versus “illness-typical” subscales of symptoms experienced, linear regression analyses modeled the association between asthma group and control group scores on each symptom subscale, controlling for demographics and pandemic-specific adverse events. These analyses revealed that people with asthma endorsed significantly more asthma-typical symptoms than controls (b = 0.76, *t*(220) = 5.96, *p* < 0.001), but not significantly more COVID illness-typical symptoms (b = 0.22, *t*(220) = 1.78, *p* = 0.076). Additionally, an exploratory analysis examining the two COVID-19 symptom subscales as mediators of the effect of asthma group on emotional exhaustion revealed that the asthma-typical subscale of COVID-19 symptoms significantly mediated the relationship between asthma group and emotional exhaustion (a*b = 1.53, 95%CI = [0.72, 2.51], *p* < 0.05, P_m_ = 0.42). The infection-typical symptom scale, however, did not mediate the effect of asthma group on emotional exhaustion (a*b = 0.32, 95%CI = [− 0.07, 0.85]) (Figure S1, Table S5a-b). Finally, both symptom subscales were significantly correlated with scores on perceived stress (PSS-4) (asthma-typical: *r*(234) = 0.27, *p* < 0.001; infection-typical: *r*(234) = 0.39, *p* < 0.001).

In sensitivity analyses, we repeated the analyses outlined above using the non-imputed data set. Similar regression coefficients and associated level of significance were seen in analyses run on imputed and non-imputed data.

Finally, we investigated additional potential confounds such as health and perceived socioeconomic status (SES). The association of asthma with emotional exhaustion remained significant in analyses for Aims 1 and 2 when different comorbid health conditions (e.g., obesity, diabetes) were added independently as covariates. Similarly, replacing years of school (as proxy for SES) with scores on the MacArthur scale of Subjective Social Status did not change the significance of results (Adler et al., 2000; Baker, 2014).

## Discussion

The present study examined the effect of asthma on various domains of psychological distress during the COVID-19 pandemic. Results provide novel insight into the differential psychological experience of people with asthma during the COVID-19 pandemic. As predicted, our findings indicate that a current diagnosis of asthma was associated with elevated scores in multiple domains of psychological distress, though emotional exhaustion was the only outcome that was significantly elevated compared to controls when correcting for FDR and when controlling for general anxious and depressive symptoms (Higbee et al., 2021).

Psychological burnout is a state of emotional, physical, and mental exhaustion associated with functional impairment in one’s professional and/or daily life. These feelings are thought to result from the increased energy demand of coping in high-stress environments, with emotional exhaustion thought to be the first domain of burnout to appear in response to prolonged stress (Aronsson et al., 2017). We found that asthma-typical symptom experience accounted for a significant portion of variance in elevated emotional exhaustion, and this mediating effect was particularly strong for those symptoms that overlapped with symptoms of COVID-19 (e.g., chest tightness). A prior study found that experience of respiratory symptoms was cited as a main source of pandemic-era stress for people with asthma (Philip et al., 2020), and thus the increased energy required to cope with symptom exacerbations in a pandemic setting could hasten a progression to burnout for those with asthma (Foster et al., 2017; McDonald et al., 2018). While it must be noted that asthma-typical symptoms may have also been present in individuals who did not endorse a current diagnosis of asthma, our results revealed that a diagnosis of asthma was significantly associated with more asthma-typical symptoms but not COVID illness-typical symptoms. This finding lends further validity to the self-reported diagnosis of asthma used to create our groups and increases confidence in the conclusion that elevated emotional exhaustion in the asthma group is preceded by their increased experience of asthma symptoms compared to controls.

When viewed from the perspective of the metacognitive theory of dyshomeostasis framework (Stephan et al., 2016), persistence of dyshomeostasis in the form of reduced asthma control would generalize into perceived lack of control and hopelessness—two characteristics of burnout. Most individuals in this study (60.6%) experienced more exacerbations during the COVID-19 pandemic than they had prior. As psychological triggers of asthma are common among people with asthma (Ritz et al., 2016), this theory may be particularly well suited for understanding how burnout can manifest because of low allostatic self-efficacy. Indeed, this theory would suggest that a perceived worsening of asthma symptoms will lead to chronic fatigue and negative beliefs about one’s ability to regulate their health, resulting in the general exhaustion and decreased motivation endorsed by the asthma group on the MBI-GS (e.g., “I feel tired when I get up in the morning and have to face another day”). Further, people with asthma endorsed experiencing “death of friend or family member due to COVID-19” significantly more often than controls. Although this item was not independently associated with MBI-GS scores, it must be considered that it may have contributed to allostatic low self-efficacy in this population by strengthening their belief that individuals are unable to control their physical health. When viewed through the lens of the metacognitive theory of dyshomeostasis framework, this additional blow to one’s perceived allostatic self-efficacy could hasten and/or intensify the top-down progression from fatigue to a state of psychological burnout.

While the distinction of burnout from other psychological disorders has been a topic of debate (Schonfeld et al., 2018), the results of this study suggest that domains of psychological burnout could manifest differently from depression and anxiety in people with asthma. It has been established that anxiety and depression symptoms are related to physical symptom burden in asthma, negatively affect asthma management, and—particularly in the case of depressive symptoms—decrease control of asthma symptoms. However, our results suggest a separate, previously unidentified state of “symptom burnout”—that is, exhaustion of emotional resources associated with a diagnosis of asthma and experience of asthma-related symptoms. Recognizing this source of emotional exhaustion related to asthma symptom experience could be particularly relevant to symptom-focused clinical management of asthma. However, it should be noted the cross-sectional nature of our study precludes firm conclusions about causality at this time.

Contrary to our expectations, neither worry about asthma-typical symptoms nor perceived COVID-19 vulnerability mediated the relation between asthma and emotional exhaustion. Rather than representing worry about contracting COVID-19, results suggest that the emotional exhaustion endorsed by people with asthma could result from symptom experience itself—an interpretation that would be supported by the metacognitive theory of dyshomeostasis. Indeed, a significant amount of the difference in emotional exhaustion between asthma and control groups was accounted for by experience of symptoms typical in asthma. While some people without a current asthma diagnosis may experience occasional asthma-typical symptoms, our results show that, unsurprisingly, people with asthma endorsed significantly more asthma symptoms than controls, although this difference was not seen in illness-typical symptom scores. It is important to consider that chronic dyshomeostasis may have been present for this group before the threat of COVID-19, as also suggested by a recent survey that revealed high levels of fatigue in patients seeking specialist care for asthma (Salsman et al., 2021). However, their allostatic self-efficacy may have been further challenged in the face of pandemic-related barriers and stress. Asthma management was suboptimal in the U.S. before the pandemic, particularly in populations subject to financial, geographical, or other barriers to healthcare, with studies reporting up to 80% of American asthma patients experiencing suboptimal symptom control, according to clinical standards. These rates are reflected in substantial health and economic burdens to the country (Marcus et al., 2008; Yaghoubi et al., 2019).

Thus far, the results of studies investigating pandemic-related stress effects on asthma control have been mixed. While adherence to asthma control measures seemed to improve at the start of the COVID-19 pandemic and ED admissions dropped (Nourazari et al., 2021, Skene & Pfeffer, 2021), studies of self-reported symptoms suggest that people with asthma were more likely to rate their symptoms as worse since the start of the pandemic—particularly if they had contracted the virus themselves (Gomes et al., 2023; Muntean et al., 2023). The ACT scores of most people in the asthma group indicated that their asthma was “well-controlled”. Given reports of worsened asthma symptom experience during the same period, higher scores may reflect elements of medication adherence and level of symptom-related functional impairment not captured by our COVID-19 symptom experience measure. This finding is precedented by literature indicating that while asthma medication adherence improved at the start of the pandemic, self-report surveys generally indicated that asthma symptoms increased during the pandemic (Gomes et al., 2023; Muntean et al., 2023; Skene & Pfeffor,^2021^). If faced with novel uncertainty, perceived vulnerability, and barriers to healthcare in a pandemic setting, the metacognitive theory of dyshomeostasis would predict that perceived symptom burden would be associated with lowered allostatic self-efficacy, fatigue, and “symptom burnout”. Results highlight the importance of self-report asthma symptom measures alongside medication adherence and disease-related functional impairment, particularly if we are to move towards a patient-centered model of care for people with asthma that considers psychological well-being an important dimension of overall health.

There are several limitations to our study that should be considered. The generalizability of our results is hindered by a self-selected sample with a higher representation of white, urban, female individuals. However, a mean age of 55.7 years was an advantage of our study, in that it represents an older adult demographic that has traditionally been underrepresented in asthma research. These patients were at risk for more severe COVID-19 symptomology, and our cross-section of psychological health 3–6 months into pandemic restrictions provided insight into potential effects of sustained versus initial, more acute stress (Teague et al., 2018). Given that our sample lacked adequate representation of those with historical barriers to asthma care (i.e., minority groups, rural populations), the barriers to care presented by the pandemic may have been novel to many participants (Forno & Celedón, 2009; Smith et al., 2009). COVID-19 disproportionately affected ethnic minorities and people living in low-SES or rural settings (Karmakar et al., 2021), and further research is needed to understand how “symptom burnout” could manifest in those who lived already with suboptimal asthma care before COVID-19 restrictions. Additionally, it is difficult to determine the specificity of the observed elevations in distress to the pandemic without a pre-COVID-19 baseline measurement, and the cross-sectional nature of the study also hinders firm conclusions about the causal nature of the relationship between the variables studied here. Finally, because of pandemic-related considerations, it was necessary to rely on self-reported asthma diagnosis and mental health conditions versus in-person medical screening. Though not ideal, self-reported diagnosis of asthma has been demonstrated to be reliable and valid by previous research, and the inclusion of a validated measure of psychopathology as a control measure (PHQ-4) makes this a more conservative approach to estimating the amount of pandemic-related distress in asthma (Iversen et al., 2007; Mirabelli et al., 2014).

## Summary and conclusion

In summary, this study provides novel insight into the differential nature of psychological distress experienced by people with asthma during the COVID-19 pandemic. Results contribute to our understanding of how symptom burden contributes to the psychological functioning of people with asthma, especially in a period of heightened environmental stress. People with asthma suffered from stronger anxiety, perceived stress, and burnout symptoms during the pandemic compared to controls, and emotional exhaustion symptoms of burnout remained significantly higher after controlling for general anxiety and depression symptoms. Our results also show that increased experience of symptoms present in both COVID-19 and asthma partially mediated the relationship between asthma and burnout symptoms. The mediating role of asthma/COVID-19 symptom experience may indicate that allocation of more energy toward coping with asthma symptoms in the setting of a national emergency may have resulted in higher emotional exhaustion in those with asthma. To our knowledge, this phenomenon has not yet been identified in the literature. As worry about symptoms indicating COVID-19 infection and perceived COVID-19 vulnerability did not alter the effect of asthma on emotional exhaustion, more research is needed into the generalizability of “symptom burnout” to non-pandemic settings. Substantial barriers to care exist for many populations outside of a pandemic, and future research could provide insight into how experience of symptoms, while lacking access to healthcare resources, could lead to emotional exhaustion. Future work could also explore the potential contribution of individual health behaviors, perceived health status, and coping styles to management of anxiety, stress, and burnout symptoms during a pandemic, as well as the relationship of fatigue to “symptom burnout”.

## Supplementary Information

Below is the link to the electronic supplementary material.

Supplementary file1 (PDF 422 kb)

## Data Availability

Full dataset available upon request to the corresponding author.

## Code availability

Full software code is available upon request to the corresponding author.
