# Supplementary material for: Psychological distress and symptom-related burnout in asthma during the COVID-19 pandemic
Source: J Behav Med. 2023 May 25;46(6):960–72. doi: 10.1007/s10865-023-00412-y (PMC10211287; doi:10.1007/s10865-023-00412-y)
Supplement: Supplementary file 1 — Supplementary file1 (PDF 422 kb) [file 10865_2023_412_MOESM1_ESM.pdf]

**Table S1.** EFA Pattern Matrix: Symptom Experience Scale		
	**Factor 1** **“Asthma-typical”** **α=.90**	**Factor 2** **“Illness-typical”** **α=.84**
1. Fever/chills	0.31	**0.51**
2. Cough	**0.62**	0.24
3. Shortness of breath	**1.07**	-0.20
4. Chest tightness	**0.94**	-0.08
5. Fatigue	**0.58**	0.17
6. Muscle/body aches	**0.46**	0.30
7. Headache	0.31	**0.37**
8. Wheezing	**0.87**	-0.11
9. New loss of taste or smell	-0.23	**0.70**
10. Sore throat	-0.06	**0.81**
11. Congestion/runny nose	0.34	**0.42**
12. Nausea/vomiting	0.08	**0.52**
13. Diarrhea	0.10	**0.53**

[1] Bolded values indicate symptom cluster grouping (>.35) of each individual symptom.
[2] EFA= Exploratory Factor Analysis with Maximum Likelihood Estimation, Promax rotated with Kaiser Normalization, α= Cronbach’s alpha of scale formed from key loading items.

Table S2. Regression model for the “b” path in the mediation model investigating **perceived COVID-19 vulnerability** as a mediator of the relationship between asthma diagnosis and pandemic-related emotional exhaustion.

	**B**	***t***	***% Variance***
**Age**	-0.08	-1.44	0.51%
**Gender**	-0.92	-0.73	0.13%
**Race**	0.80	0.67	0.11%
**Years of Education**	0.29	1.49	0.52%
**Pandemic-specific adverse events**			
Loss of employment	-0.32	-0.28	0.02%
Inability to pay bills	-1.58	-1.16	0.31%
Child in home needing care	-0.08	-0.06	0.00%
Increase in home conflict	-0.18	-0.14	0.00%
Relocated home	2.15	1.09	0.27%
Improvise living conditions	0.53	0.38	0.03%
Unable to get food	-3.15	-1.81	0.75%
Death of close friend or family member	-2.20	-1.37	0.44%
**PHQ-4 - Anxiety**	1.99	**5.00*****	6.21%
**PHQ-4 - Depression**	1.65	**4.53*****	5.11%
**Asthma Group**	3.64	**3.71*****	3.28%
**COVID-19 Vulnerability**	0.13	0.50	0.06%

[1] Bolded values are significant at *p < .05, **p < .01, ***p < .001.
[2] *P*-values for the associations of asthma group, PHQ-4 Anxiety, and PHQ-4 Depression with MBI-GS Emotional Exhaustion scores remained significant after correction for multiple tests, (FDR critical value = p<.003)
[3] % Variance = percent variance over and above other variables.
[a] 0 = non-asthmatic control, 1 = asthma. [b] 0=female, 1=male. [c] 0=white, 1=not white

Table S3. Regression model for the "b" path in the mediation model investigating **COVID-19 symptom experience, worry about symptoms, and the interaction of symptom experience and worry** as a mediator of the relationship between asthma diagnosis and pandemic-related emotional exhaustion.

	**B**	***t***	***% Variance***
**Age**	-0.07	-1.22	0.35%
**Gender**	-0.72	-0.58	0.08%
**Race**	1.14	0.98	0.22%
**Years of Education**	0.35	1.88	0.77%
**Pandemic-specific adverse events**			
Loss of employment	-0.85	-0.76	0.12%
Inability to pay bills	-1.71	-1.29	0.36%
Child in home needing care	0.32	0.24	0.01%
Increase in home conflict	-0.81	-0.65	0.09%
Relocated home	2.04	1.06	0.24%
Improvise living conditions	0.42	0.31	0.02%
Unable to get food	-3.05	-1.81	0.71%
Death of close friend or family member	-1.68	-1.08	0.25%
**PHQ-4 - Anxiety**	1.89	**4.87*****	5.48%
**PHQ-4 - Depression**	1.48	**4.20*****	4.12%
**Asthma Group**	2.63	**2.58****	1.51%
**Symptom Experience**	1.47	**1.97***	0.96%
**Symptom Worry**	0.52	0.99	0.24%
**Interaction Experience*Worry**	-0.27	-0.68	0.11%

[1] Bolded values are significant at $^{*}p < .05$, $^{**}p < .01$, $^{***}p < .001$.
[2] *P*-values for the associations of PHQ-4 Anxiety and PHQ-4 Depression with MBI-GS Emotional Exhaustion scores remained significant after correction for multiple tests, (FDR critical value = p<.003)
[3] % Variance = percent variance over and above other variables.
[a] 0 = non-asthmatic control, 1 = asthma. [b] 0=female, 1=male. [c] 0=white, 1=not white

8

Table S4. Regression model for the "b" path in the mediation model investigating **asthma control** as a mediator of the relationship between asthma diagnosis and pandemic-related emotional exhaustion.

	**B**	***t***	***% Variance***
**Age**	-0.10	-1.21	1.08%
**Gender**	1.83	0.73	0.40%
**Race**	-0.09	-0.05	0.00%
**Years of Education**	0.30	1.12	0.79%
**Pandemic-specific adverse events**			
Loss of employment	0.59	0.35	0.08%
Inability to pay bills	-0.96	-0.50	0.15%
Child in home needing care	-0.45	-0.24	0.04%
Increase in home conflict	1.00	0.56	0.19%
Relocated home	0.80	0.29	0.05%
Improvise living conditions	0.01	0.00	0.00%
Unable to get food	-3.02	-1.30	1.04%
Death of close friend or family member	-2.29	-1.07	0.72%
**PHQ-4 - Anxiety**	1.77	**3.05****	5.95%
**PHQ-4 - Depression**	1.17	**2.30***	3.42%
**ACT Score**	-0.34	-1.94	2.34%

[1] Bolded values are significant at **p* < .05, ***p* < .01, ****p* < .001.
[2] No *p*-values for any associations investigated above remained significant after correction for multiple tests, (FDR critical value = *p*<.003)
[3] % Variance = percent variance over and above other variables.
[a] 0 = non-asthmatic control, 1 = asthma. [b] 0=female, 1=male. [c] 0=white, 1=not white

Table S5a. Regression model for the "b" path in the mediation model investigating **asthma-typical symptoms** as a mediator of the relationship between asthma diagnosis and pandemic-related emotional exhaustion.

	**B**	***t***	***% Variance***
**Age**	-0.08	-1.53	0.53
**Gender**	-0.71	-0.58	0.08
**Race**	1.07	0.93	0.19
**Years of Education**	0.37	1.99	0.86
**Pandemic-specific adverse events**			
Loss of employment	-0.83	-0.75	0.12
Inability to pay bills	-1.76	-1.34	0.38
Child in home needing care	0.13	0.10	0.00
Increase in home conflict	-0.63	-0.51	0.06
Relocated home	2.02	1.06	0.24
Improvise living conditions	0.34	0.25	0.01
Unable to get food	-2.88	-1.73	0.64
Death of close friend or family member	-1.58	-1.02	0.22
**PHQ-4 - Anxiety**	1.80	**4.66***	5.02
**PHQ-4 - Depression**	1.51	**4.31***	4.33
**Asthma Diagnosis**	2.14	**2.12***	0.98
**Asthma-Typical Symptoms**	2.16	**4.17***	3.88

[1] Bolded values are significant at *$p < .05$, **$p < .01$, ***$p < .001$.
[2] *P*-values for the associations of Asthma-Typical Symptoms, PHQ-4 Anxiety, and PHQ-4 Depression with MBI-GS Emotional Exhaustion scores remained significant after correction for multiple tests, (FDR critical value = $p<.003$)
[3] % Variance = percent variance over and above other variables.
[a] 0 = non-asthmatic control, 1 = asthma. [b] 0=female, 1=male. [c] 0=white, 1=not white

Table S5b. Regression model for the "b" path in the mediation model investigating **infection-typical symptoms** as a mediator of the relationship between asthma group and pandemic-related emotional exhaustion.

	B	*t*	*% Variance*
**Age**	-0.06	-1.16	0.31
**Gender**	-0.55	-0.44	0.04
**Race**	1.26	1.07	0.27
**Years of Education**	0.32	1.67	0.62
**Pandemic-specific adverse events**			
Loss of employment	-0.59	-0.52	0.06
Inability to pay bills	-1.53	-1.14	0.29
Child in home needing care	0.20	0.15	0.00
Increase in home conflict	-0.64	-0.51	0.06
Relocated home	1.98	1.02	0.23
Improvise living conditions	0.32	0.24	0.01
Unable to get food	-3.14	-1.85	0.76
Death of close friend or family member	-2.01	-1.28	0.36
**PHQ-4 - Anxiety**	1.94	**4.96*****	5.90
**PHQ-4 - Depression**	1.59	**4.48*****	4.84
**Asthma Group**	3.35	**3.48*****	2.76
**Infection-Typical Symptoms**	1.58	**3.02****	2.16

[1] Bolded values are significant at *$p < .05$, **$p < .01$, ***$p < .001$.
[2] *P*-values for the associations of asthma group, Infection-Typical Symptoms, PHQ-4 Anxiety, and PHQ-4 Depression with MBI-GS Emotional Exhaustion scores remained significant after correction for multiple tests, (FDR critical value = p<.003)
[3] % Variance = percent variance over and above other variables.
[a] 0 = non-asthmatic control, 1 = asthma. [b] 0=female, 1=male. [c] 0=white, 1=not white

TABLE S6. FULL LIST OF COVID-19 SPECIFIC ADVERSE EVENTS ITEMS.

INSTRUCTIONS: FOR EACH STATEMENT BELOW, PLEASE INDICATE WHETHER THE CORONAVIRUS-19 PANDEMIC HAS IMPACTED YOU AND/OR MEMBERS OF YOUR HOUSEHOLD IN THE WAY DESCRIBED. IF BOTH YOU AND A MEMBER OF YOUR HOUSEHOLD HAVE BEEN AFFECTED IN THE WAY DESCRIBED, PLEASE CHECK BOTH YES (ME) AND YES (PERSON IN HOME).

ANSWER CHOICES: 0, NO | 1, YES, ME

1. LAID OFF FROM JOB, HAD TO QUIT JOB, OR HAD TO CLOSE OWN BUSINESS.*
2. HAD TO CONTINUE TO WORK EVEN THOUGH IN CLOSE CONTACT WITH PEOPLE WHO MIGHT BE INFECTED (E.G., CUSTOMERS, PATIENTS, CO-WORKERS).
3. DIFFICULTY MAKING THE TRANSITION TO WORKING FROM HOME.
4. UNABLE TO PAY IMPORTANT BILLS LIKE RENT OR UTILITIES.*
5. HAD A CHILD IN HOME WHO NEEDED YOUR CARE.*
6. INCREASE IN CONFLICT WITH A PARTNER OR SPOUSE.
7. INCREASE IN CONFLICT BETWEEN MEMBERS OF THE HOUSEHOLD.*
8. FAMILY OR FRIENDS HAD TO MOVE INTO YOUR HOME.
9. HAD TO MOVE OR RELOCATE.*
10. BECAME HOMELESS.
11. HAD TO IMPROVISE LIVING CONDITIONS.*
12. DID NOT HAVE ENOUGH PERSONAL SPACE WHERE I LIVED.
13. DID NOT HAVE THE ABILITY OR RESOURCES TO SEE FAMILY OR FRIENDS WHILE SEPARATED.
14. DID NOT HAVE THE ABILITY OR RESOURCES TO TALK TO FAMILY OR FRIENDS WHILE SEPARATED.
15. RELIGIOUS OR SPIRITUAL ACTIVITIES CANCELLED OR RESTRICTED.
16. UNABLE TO ATTEND IN-PERSON SERVICES (I.E. FUNERAL, WEDDING) FOR FAMILY MEMBERS.
17. UNABLE TO PERFORM ENJOYABLE ACTIVITIES OR HOBBIES.
18. UNABLE TO GET ENOUGH FOOD OR HEALTHY FOOD.*
19. UNABLE TO ACCESS CLEAN WATER.
20. DIFFICULTY GETTING PLACES DUE TO LESS ACCESS TO PUBLIC TRANSPORTATION OR CONCERNS ABOUT SAFETY.
21. ISOLATED OR QUARANTINED DUE TO POSSIBLE EXPOSURE TO THIS DISEASE.
22. ISOLATED OR QUARANTINED DUE TO SYMPTOMS OF THIS DISEASE.
23. ISOLATED OR QUARANTINED DUE TO EXISTING HEALTH CONDITIONS THAT INCREASE RISK OF INFECTION OR DISEASE.

24. ISOLATED OR QUARANTINED DUE TO PRIMARY CAREGIVER DUTY FOR A VULNERABLE PERSON.
25. TESTED AND CURRENTLY HAVE CORONAVIRUS-19.
26. TESTED POSITIVE FOR CORONAVIRUS-19 BUT NO LONGER HAVE IT.
27. CURRENTLY HAVE OR HAVE HAD SYMPTOMS OF CORONAVIRUS-19 BUT NEVER TESTED.
28. HAVE SOUGHT TESTING FOR CORONAVIRUS-19 BUT HAVE NOT HAD ACCESS TO A TEST.
29. HOSPITAL STAY DUE TO CORONAVIRUS-19.
30. DEATH OF CLOSE FRIEND OR FAMILY MEMBER FROM CORONAVIRUS-19.*

** Item was endorsed by >10% of the sample and included as a control variable in multiple regression analyses.*

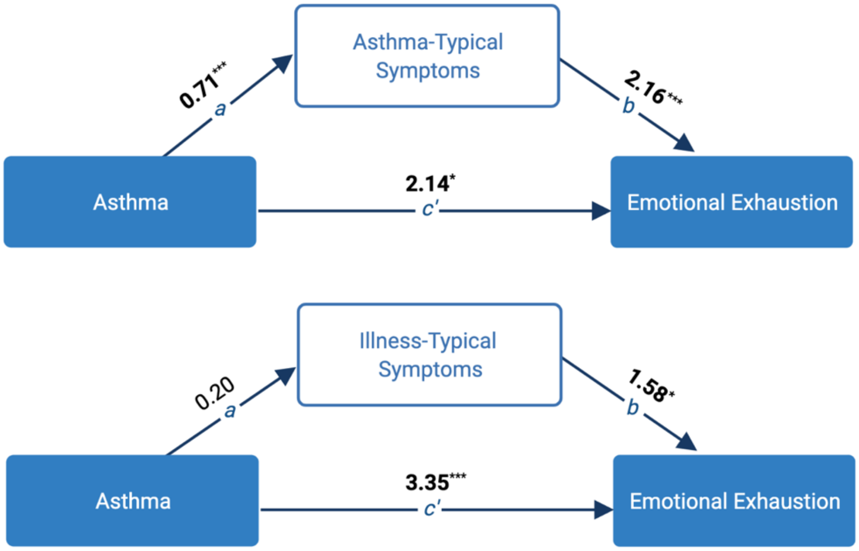

Figure S1. Mediation effect of the two categories of symptoms derived from the Symptom Experience Scale on the relationship between asthma and emotional exhaustion. Symptoms that overlap between COVID-19 and asthma significantly mediated the main effect of asthma (P_m=.42), but viral illness-specific symptoms did not. *Significant at *P*< .05, ***Significant at *P*< .001.
